# Supplementary material for: Perspectives of wheelchair users with spinal cord injury on fall circumstances and fall prevention: A mixed methods approach using photovoice
Source: PLoS One. 2020 Aug 28;15(8):e0238116. doi: 10.1371/journal.pone.0238116 (PMC7454945; doi:10.1371/journal.pone.0238116)
Supplement: S1 File — (DOCX) [file pone.0238116.s001.docx]

**Supplementary Material: Fall survey**

**Fall and Near-Fall Survey**

Please complete this survey within 24 hours of having a fall or near-fall. One survey should be completed for each fall/near-fall. A fall is “an event which results in a person coming to rest inadvertently on the ground or floor or other lower level” (World Health Organization 2013). A near-fall is “a loss of balance in which the person starts to fall but is able to catch himself or herself before landing” (Ryan et al. 1993).

1. Participant code: __________
2. I had a (please select one): Fall □ Near-fall □
3. Date of fall/near-fall: __________
4. At what time of day did you have the fall/near-fall? Please select one:

Morning □ Afternoon □ Evening □ Night □

1. Where did you have the fall/near-fall? Please select one:

Home indoors □ Home outdoors □

Community indoors □ Community outdoors □

Work indoors □ Work outdoors □

1. What were you doing when you had the fall/near-fall? Please select one and explain further. For example, if you tripped on a throw rug while walking, check off ‘Walking’ and write “tripped on throw rug” after ‘Details’.

Standing □ Details_________________________________________

Changing positions (e.g., sitting to standing) □ Details__________________

Walking □ Details________________________________________

Climbing stairs □ Details_________________________________

Getting into/out of bed □ Details______________________________

Getting into/out of shower/bath □ Details____________________________

Getting into/out of a vehicle □ Details____________________________

Opening/closing a door □ Details____________________________

Other □ Details_________________________________________________

1. Why do you think you had this fall/near-fall? Please select all that apply to this fall/near-fall:

Tripped □ Slipped □

Was distracted □ Doing more than 1 thing (e.g., walk & talk) □

Poor balance □ Legs gave out □

Weakness in my legs □ Felt dizzy □

Weather (e.g., rain, ice) □ Moving quickly/rushing □

Tired □ Not using my walker/cane/brace □

Spasms in my legs □ Dark/poorly lit environment □

Problems with my vision □ Alcohol/drug consumption □

Don’t know □ Illness □

Other □ Details_______________________________________________

1. Were you injured as a result of this fall/near-fall? Yes □ No □

If yes, what was your injury? Please select all that apply:

Bruises □ Broken bone □ Where?___________________

Cuts/scrapes □ Joint dislocation □ Where?_________________

Bumped head □ Pain □ Where?______________________

Other □ Details______________________________________________

1. Did you seek medical attention because of this fall/near-fall? Yes □ No □
2. Where you admitted to the hospital as a result of this fall? Yes □ No □
3. Was there a recent change in your medications? For example, did you start taking a new drug, stop taking a drug or change the dosage of a drug? Yes □ No □

If yes, please provide details: _____________________________________________
