# Supplementary material for: Perspectives of wheelchair users with spinal cord injury on fall circumstances and fall prevention: A mixed methods approach using photovoice
Source: PLoS One. 2020 Aug 28;15(8):e0238116. doi: 10.1371/journal.pone.0238116 (PMC7454945; doi:10.1371/journal.pone.0238116)
Supplement: S2 File — (DOCX) [file pone.0238116.s002.docx]

Supplementary material B: Focus group interview guide

**Discussion of trends from the fall surveys**

More than half of total wheelchair users in this study fell at least once in 6 months. What are your thoughts on this?

Discussion of time and location of falls (e.g. most falls happened in the home during the afternoon and evening. Why do you think that is?)

Discussion of activities during falls (e.g. many falls for manual wheelchair users happened while wheeling over ground, transfers, during sports/exercise and walking/standing. Falls for power wheelchair users happened during transfers and driving through doorways. Why do you suppose this is?)

Discussion of common causes of falls (e.g. perceived contributors were weakness in legs, legs giving out, moving too quickly/rushing, assistant error, multi-tasking. What are your thoughts about these contributing factors?)

**Sharing photographs**

Each participant will discuss one photograph they captured that relates to factors that influence their fall risk or how the risk of falls impacts their work/leisure activities.

**Discussion of themes from individual interviews**

Now we will discuss the themes we found from the individual interviews.

Theme 1: Individualizing and customizing fall prevention

This theme reflects the lived experience/individualized/personalized experience with fall prevention, which each participant spoke about. For many, fall prevention was individualized to their 1) specific level of injury, 2) their daily activities, 3) their environment (home and community), 4) level of support they have, and 5) current health status which largely influences their level of function (muscle strength, tolerances for activities, level of assistance needed). What are your thoughts on this?

Theme 2: Aging with spinal cord injury impacts fall risk

Aging increases fall risk in terms of difficulty of transfers, over-use injuries, greater impact of falls (risk of fractures). What are your thoughts on this?

Theme 3: Impact of Falls

Falls impact peoples everyday lives such as with work, parenting, social and recreation. Could you think about some examples of how the risk of falling impacts your everyday life and share this information with us? What are your thoughts on this?

Theme 4: Peer mentorship approach to fall prevention education

Those with lived experience of falls were perceived as useful resources to develop fall prevention strategies. What are your thoughts on this?

**Discussion informed by themes from individual interviews**

1. Are falls preventable? For those that said yes, how can they be best prevented? For those that said no, why is that?
2. Could you share your most useful strategies to prevent falls?
   1. Probe: What are the top things that decrease your risk of falling? E.g anti-tippers (how many have these on), seat belt (how many wear it regularly)
3. Can you share with us how falls impact your life?
   1. Probe: How concerned are you about falls in your everyday life?
   2. How do falls impact your participation in daily activities?
   3. How do falls impact parenting?
   4. How do falls impact your mobility? (indoors, outdoors)
   5. How do falls impact your life satisfaction?
   6. Social life?
   7. Can you give me an example?
4. Please describe the most useful fall prevention strategies or advice you have received?
   1. Probe: Who provided the training?
   2. Can someone build on that?
5. How does the environment impact your risk of falling?
   1. Probe: What are the top three things that increase your risk of falling?
   2. How does equipment impact risk of falling? (new equipment)
   3. How does the weather impact your risk of falling
6. In your opinion, in what ways does a spinal cord injury impact your fall risk?
7. Are there different types of falls (e.g. ones you can anticipate more while others are unpredictable ones)
   1. How does aging with SCI impact your fall risk?
8. What are important things for clinicians to know about falls and fall prevention after SCI?

**Designing a fall prevention class**

For this activity, as a group, we will design an ‘ideal’ fall prevention class for people with SCI. Feel free to share what you think it should look like.

1. Who would be involved? Who should lead it?
2. When should it happen?
3. Where should it happen?
4. How long should it be? How should the structure be?
5. What would it include? Why is it important to include this?
6. Please describe the most useful fall prevention strategies or advice you have received?
   1. Probe: Who provided the training?
7. What are the things or situations that most likely increase your fall risks?
8. How does the environment impact your risk of falling?
   1. Probe: What are the top three things that increase your risk of falling?
9. How does your spinal cord injury impact your fall risk?
10. What advice would you give to others with SCI about fall prevention?
11. What are important things for clinicians to know about falls and fall prevention after SCI?

**Knowledge translation**

You have all worked so hard to take these pictures and we have great information on fall prevention. How would you like to present this data?
