# Supplementary material for: Perspectives of wheelchair users with spinal cord injury on fall circumstances and fall prevention: A mixed methods approach using photovoice
Source: PLoS One. 2020 Aug 28;15(8):e0238116. doi: 10.1371/journal.pone.0238116 (PMC7454945; doi:10.1371/journal.pone.0238116)
Supplement: S3 File — (DOCX) [file pone.0238116.s003.docx]

S3: Good Reporting of A Mixed Methods Study (GRAMMS)

| **Component** | **Section/Pages** |
| --- | --- |
| Describe the justification for using a mixed methods approach to the research question | Introduction pages 5-6  Methods (design) page 6 |
| Describe the design in terms of the purpose, priority and sequence of methods | Methods (design) page 6  Methods (data collection) pages 8-9 |
| Describe each method in terms of sampling, data collection and analysis | Methods page 6-10 |
| Describe where integration has occurred, how it has occurred and who has participated in it | Figure 1  Methods (data collection and analysis) pages 8-10 |
| Describe any limitation of one method associated with the present of the other method | Discussion pages 32-33 |
| Describe any insights gained from mixing or integrating methods | Discussion page 33 |

O'Cathain A, Murphy E, Nicholl J. The quality of mixed methods studies in health services research. J Health Serv Res Policy. 2008;13(2):92-8. Epub 2008/04/18. doi: 10.1258/jhsrp.2007.007074. PubMed PMID: 18416914.
